# Differential influence of race and environment on indeterminate reactivities to non-treponemal and treponemal antigens by immuno-chromatographic dual syphilis rapid test

**DOI:** 10.11604/pamj.2019.33.90.16437

**Published:** 2019-06-06

**Authors:** Francois-Xavier Mbopi-Keou, Ginette Claude Mireille Kalla, Esther Voundi Voundi, Mohammad-Ali Jenabian, Ralph-Sydney Mboumba Bouassa, Frédéric Talla, Fru F Angwafo, Laurent Belec

**Affiliations:** 1 University of Yaounde I, Faculty of Medicine and Biomedical Sciences, Yaoundé, Cameroon; 2 The Institute for the Development of Africa (The-IDA), Yaoundé, Cameroon; 3 UNAIDS Scientific and Technical Advisory Committee (STAC); 4 Department of Biological Sciences and BioMed Research Centre, Université du Québec à Montréal (UQAM), Montréal, Québec, Canada; 5 Laboratoire de Virologie, Hôpital Européen Georges Pompidou, Assistance Publique-Hôpitaux de Paris, Paris, France; 6 Laboratoire Litto-Labo, Douala, Cameroon; 7 Gynecologic and Pediatric Hospital, Yaoundé, Cameroon; 8 Faculté de Médecine Paris Descartes, Université Paris Descartes (Paris V), Sorbonne Paris Cité, Paris, France

**Keywords:** Syphilis, immuno-chromatographic rapid test, false positivity, race, environment, Central Africa, France

## Abstract

**Introduction:**

Syphilis rapid test results may be influenced by numerous environmental and genetic factors.

**Methods:**

The proportion of false positive syphilis non-treponemal (NT) and treponemal (T) test results using immuno-chromatographic dual syphilis rapid test on serum from Cameroonian blacks (n=103) versus French blacks (n=104) or French caucasians (n=51), all HIV-negative and free of clinical syphilis, was examined.

**Results:**

Black individuals in Cameroon had a significantly higher frequency of false positive NT or T tests than black individuals in France. black individuals in France had a higher frequency of indeterminate NT tests as compared to caucasians in France.

**Conclusion:**

Both racial and environmental factors may affect immuno-chromatographic dual syphilis rapid testing.

## Introduction

Syphilis rapid test results may be influenced by numerous environmental and genetic factors [1-6]. We herein report on the frequency of false-positive non-treponemal (NT) and treponemal (T) tests using a dual syphilis rapid test on serum from black individuals living in Central Africa versus black and caucasian individuals living in France.

## Methods

Collection of serum aliquots (conserved at -20°C) from routine serological testing according to medical prescription of black adults in Cameroon, and black and caucasian adults in France, as controls, was used, in agreement with institutional ethical and research review boards of Laboratoires Litto Labo, Hygiene Mobile in Cameroon [7, 8] and Assistance Publique de Paris (processing number 1922081) in France. All selected individuals had given their oral informed consent for sampling, were HIV-negative and free of any clinical manifestations of syphilis. No personal identifiers were collected.

All sera selected for the study were negative by reference gold standard NT and T syphilis serology, including RPR test for NT antibodies (Arlington Scientific Inc., Springville, Utah, USA), and ELISA test for T. pallidum-specific IgG+IgM antibodies (DiaSorin, Vercelli, Italy). The rapid point-of-care immuno-chromotographic dual test RDT DPP^®^ Syphilis Screen & Confirm Assay (Chembio Diagnostics Systems Inc., Medford, NY, USA) was used for simultaneous detection of both NT and T antibodies, as described [9, 10]. Readings were made independently by two technicians exactly 15 minutes after the addition of the last running buffer. For one given line, concordant interpretations gave the final results, e.g. negative, positive or doubtful.

## Results

A total of 103 sera were prospectively collected in Cameroon, and 155 sera in France, from 104 blacks and 51 caucasians. All sera negative by the reference gold standard for syphilis serology were further tested by the syphilis rapid test. Indeterminate NT reactivities by syphilis rapid test on sera from individuals from Central Africa (23.3%) were more frequent than that observed on sera from black and caucasian individuals living in France (12.5% and 1.9%, respectively) (P<0.05 and P<0.0004, respectively) (Table 1). The prevalences of indeterminate NT reactions were high in black than caucasian people living in France (P<0.04).

**Table 1:** Non-treponemal and treponemal reactivities by the immuno-chromatographic test TDR DPP^®^ Syphilis Screen & Confirm Assay (Chembio Diagnostics Systems Inc., Medford, NY, USA)

	Cameroonian black	French black	French caucasian	*P^c^*
n	103	104	51	
**Nontreponemal reactivity** (*n;* %)				
Reference serology, RPR ^a^	0 (0.0)	0 (0.0)	0 (0.0)	
Dual rapid test, nontreponemal line	24 (23.3)	13 (12.5)	1 (1.9)	
Cameroonian Black *versus* French Black				<0.05
Cameroonian Black *versus* French Caucasian				<0.0004
French Black *versus* French Caucasian				<0.04
**Treponemal reactivity** (*n;* %)				
Reference serology, IgG+IgM ELISA^b^	0 (0.0)	0 (0.0)	0 (0.0)	
Dual rapid test, treponemal line	10 (9.7)	2 (1.9)	0 (0.0)	
Cameroonian Black *versus* French Black				<0.02
Cameroonian Black *versus* French Caucasian				<0.04
French Black *versus* French Caucasian				NS
**Indeterminate syphilis reactivities** (*n;* %)^d^	34 (33.0)	15 (14.4)	1 (1.9)	
Cameroonian Black *versus* French Black				<0.002
Cameroonian Black *versus* French Caucasian				<0.05
French Black *versus* French Caucasian				<0.04
**Active syphilis false positivity** (*n;* %)^e^	9 (8.7)	2 (1.9)	0 (0.0)	
Cameroonian Black *versus* French Black				<0.04
Cameroonian Black *versus* French Caucasian				<0.04
French Black *versus* French Caucasian				NS

a The reference serology for non-treponemal line was the Rapid Plasma Reagin test (Arlington Scientific Inc., Spingville, Utah, USA); sera whose RPR titers were ≥ 1:2 were considered as positive;

b The reference serology for the treponemal line was the indirect ELISA for *Treponema pallidum*-specific IgG and IgM antibodies directed to 15-, 17-, and 47-kDa recombinant proteins as antigens (DiaSorin, Vercelli, Italy);

c Statistical analyses were accrued out using Fisher exact test using GraphPad Prims software (version 5, La Jolla, CA, USA);

d Indeterminate syphilis reactivities was defined as one or two bands by the immune-chromatographic dual test TDR DPP^®^ Syphilis Screen & Confirm Assay ;

e Active syphilis false positivity was defined as two positive bands by the immune-chromatographic dual test TDR DPP^®^ Syphilis Screen & Confirm Assay.

n: Number; NS: Not significant

Treponemal T reactivities by syphilis rapid test on sera from individuals from Central Africa (9.7%) were more frequent than that observed on sera from black and caucasian individuals living in France (1.9% and 0.0%, respectively) (P<0.02 and P<0.04, respectively), whereas the prevalences of indeterminate T reactivities were similar in black and caucasian people living in France. When considering all NT or T reactivities, sera from individuals from Central Africa were more frequently indeterminate by syphilis rapid test than sera from black and caucasian individuals living in France. Furthermore, black individuals living in France were more frequently indeterminate by dual syphilis rapid test than caucasian individuals living in France.

Finally, when considering double NT and T reactivities, sera from individuals from Central Africa were more frequently false positive by syphilis rapid test than sera from black and caucasian individuals living in France, whereas the prevalences of indeterminate NT and T reactivities were similarly low in black and caucasian people living in France.

## Discussion

In the present study, false positive NT or T reactivities as well as indeterminate syphilis results by immuno-chromatographic dual syphilis rapid test in individuals without a clinical history of syphilis and syphilis-negative by reference serology were more frequently observed in black individuals living in Central Africa than in black individuals living in France and in caucasian individuals living in France. Furthermore indeterminate NT reactivities by dual syphilis rapid test were more frequently observed in black individuals living in France than caucasian individuals living in France. The risk of false positive dual syphilis rapid test with positive NT and T bands was significantly higher in black individuals living in Central Africa than in black and caucasian individuals living in France, while black and caucasian individuals living in France showed similarly low risk of false positivity by rapid test. These observations point that both racial and environmental factors may affect the results of the immuno-chromatographic dual syphilis rapid test.

Similar to HIV, false positive syphilis rapid test reactions can occur due to febrile illnesses, immunizations, pregnancy, connective tissue disease and malignancy [11]. In addition, false positive syphilis reactions may also be observed in the context of immune activation occurring during malaria, hepatitis C, Chagas disease, tuberculosis and leprosy [11]. Environmental, hygienic and dietary factors may furthermore contribute to immune activation and polyclonal antibody production [7]. Genetic variability may finally account for differences in the frequency of doubtful test results. Africans have more HLA diversity and class II haplotypes as compared with other ethnic groups including caucasians [4, 6, 7], resulting in varying immunological responses to non-HIV infectious diseases and thus the nature and frequency of cross-reactive antibodies [7, 12, 13].

## Conclusion

Taken together, our observations emphasize the absolute need for rapid tests to undergo evaluation in the specific environments in which they will be deployed.

### What is known about this topic

- Syphilis rapid test results may be influenced by numerous environmental and genetic factors.

### What this study adds

- Both racial and environmental factors may affect immuno-chromatographic dual syphilis rapid testing;
- Our observations emphasize the absolute need for rapid tests to undergo evaluation in the specific environments in which they will be deployed.

## Competing interests

The authors declare no competing interests.
